# Self-reported concussion prevalence, post-injury help-seeking behaviour, and associated risk factors among volleyball players

**DOI:** 10.1371/journal.pone.0338225

**Published:** 2025-12-05

**Authors:** Gamze Nedzhipoglu, Amanda Johnson, Nick Dobbin

**Affiliations:** Department of Health Professions, Faculty of Health and Education, Manchester Metropolitan University, Manchester, United Kingdom.; İzmir Democracy University: Izmir Demokrasi Universitesi, TÜRKIYE

## Abstract

**Background:**

Volleyball is an under-researched sport, particularly in relation to risk factors for head impacts and sports-related concussion (SRC). This study aimed to estimate the self-reported lifetime prevalence of SRC, post-injury help-seeking behaviour, and associations between SRC and age, sex at birth, playing experience, playing league, and playing position.

**Methods:**

A cross-sectional study was conducted using an online questionnaire to examine lifetime prevlaance and associations between SRC and personal and sport-related factors. The questionnaire collected demographic information, SRC history, and help-seeking behaviour. Descriptive statistics were used to estimate SRC prevalence and help-seeking behaviours among players with a history of SRC. A mixed-effect binary logistic model was conducted at a univariable and multivariable level to assess associations between SRC with playing experience, league, position, age, and gender.

**Results:**

A total of 74 volleyball players, 44 females and 30 males, completed the questionnaire. The estimated self-reported lifetime prevalence of SRC was 35% (males 33%, females 36%). Medical support was limited both during and following a SRC. Only 39% of players reporting a SRC were diagnosed, with just 23–27% seeking medical help. Older and more experienced athletes had higher odds of reporting SRC. Those aged 28 years and above showed greater odds (OR=4.58–8.91) of reporting a SRC compared to those aged 16–21, and those with more than 13 years of experience showed greater odds (OR=4.04–9.51) than those with 1–3 years’ experience. Professional players showed reduced odds (OR=0.05) compared to high school and university players in the multivariable model.

**Conclusions:**

SRC occur in volleyball with a lifetime prevalence of 35%, yet many incidents go undiagnosed and unmanaged. Older, more experienced players had greater odds of reporting a SRC, while professional-level athletes showed reduced odds when all factors are considered. Increased awareness and appropriate guidance are needed across all levels in volleyball.

## Introduction

Volleyball is a court-based sport involving coordinated limb and trunk movements during striking, blocking, and defending, which can lead to various acute and chronic injuries [1–5]. The sport includes several positions, right and left hitters, centre hitters, setters, and liberos, each with distinct responsibilities that may contribute to variations in injury patterns [1,6], particularly for common injuries involving the shoulder, knee, finger, and ankle [1,5,7–9]. Sport-related concussion (SRC) is also a notable concern in volleyball, accounting for 15–19% of all injuries [7], and has a cumulative incidence of approximately 7.1 per 100 athletes [10]. SRCs are traumatic brain injuries caused by a direct blow to the head, neck, or body during sport transmitting an impulsive force to the brain [11]. This initiates a complex neurological and metabolic cascade that can produce a range of signs and symptoms, which may appear immediately or develop over minutes to hours after the incident [11]. Although volleyball is often perceived as a low-contact sport, SRCs are increasingly recognised as a risk. Among female National Collegiate Athletic Association (NCAA) volleyball players, SRC incidence rates range from 4.69 to 7.07 per 10,000 athlete-exposures (AEs), with higher rates during competition (6.05 per 10,000 AEs) than practice (4.43 per 10,000 AEs) [2,7]. Data from the National Electronic Injury Surveillance System (2012–2021) indicate that head injuries are the second most common reason for volleyball-related emergency department visits, with SRC representing just over 50% of these cases [3]. Additionally, a retrospective study by Ezzat et al. [12] found that adolescent volleyball players missed an average of 3.1 days per season due to SRC. Together, these findings highlight that SRCs can occur during high-intensity game-play as well as in other volleyball-specific contexts.

Ball-to-head contact is the most common SRC mechanism in volleyball, followed by player-to-player collisions and ground impacts [2,9,10,12]. Mechanisms differ by sex, with SRCs in male players often associated with player contact, whereas in female players, ball contact is more frequently implicated [2,13]. Among junior athletes, females are also more likely than males to experience SRCs [14,15]. This pattern aligns with findings from Chandran et al. [16], who analysed SRC data from 100 high schools across nine sports and found that girls had higher concussion rates than boys across several sports, including basketball, lacrosse, and soccer. These findings suggest sex may influence SRC risk and associated contact patterns, warranting further investigation.

Other contextual factors such as playing level, position, and experience also appear to influence SRC risk and warrant consideration by medical professionals, sport scientists, and coaches. Injury surveillance during International Volleyball Federation tournaments from 2010 to 2014 showed that senior players had a higher injury frequency than juniors and were twice as likely to sustain time-loss injuries [13]. Most injuries occurred during match-play (62.5%), with centre players identified as particularly vulnerable [5]. Sandler et al. [3] reported that college-level athletes were more than twice as likely to sustain SRCs than high school players, potentially reflecting increased ball velocity at higher levels of play, especially during serves [17]. Together, these findings suggest that age, playing level, experience, and position may contribute to SRC risk in volleyball, though further research is needed.

Despite these insights, research on factors influencing SRC in indoor volleyball remains limited compared to other team sports. Given the potential impact of SRCs on individual performance, team dynamics, and long-term outcomes [11], it is essential to examine both the contributing factors and the nature of medical management provided. This study aims to explore the prevalence of SRC, identify factors influencing the help-seeking behaviour around SRCs, and assess the medical management of SRCs in indoor volleyball. A clearer understanding of these elements may clarify their combined influence on head injury risk and inform evidence-informed strategies for safer play.

Using an exploratory, cross-sectional research design, this study addresses the following research questions: 1) What is the estimated prevalence of self-reported SRC? 2) What are the post-injury help-seeking behaviours following a SRC? 3) Is there an association between SRC and age, sex at birth, playing experience, playing league, and playing position?

## Methods

### Study design

A cross-sectional study design using a questionnaire was employed to estimate lifetime prevalence and explore potential associations between various factors and reporting SRC. Data were collected between June and August 2024 and are reported in accordance with STROBE guidelines [18]. Ethical approval was granted by the Faculty of Health and Education Research Ethics Committee at Manchester Metropolitan University through EthOS (Ref: 67024).

### Participants

Participants were recruited through convenience sampling and snowball sampling. Stakeholders who acted as dissemination points facilitated access to the target population (i.e., volleyball players). Information was obtained from National Volleyball Federation websites and volleyball league club directories. Once collated, and to encourage collaboration, an invitation letter and a poster outlining the study objectives were emailed. In addition, Instagram and Facebook were used as social media platforms to reach potential participants when email communication was unavailable.

Eligible participants were individuals aged 16 years or older who had competed in volleyball for at least one year. Participants with a history of migraines or post-traumatic stress disorder (PTSD) were advised not to participate to minimise the risk of symptom overlap that could confound the identification of sport-related concussions (SRCs). Both migraines and PTSD can present with symptoms such as headaches, dizziness, cognitive difficulties, or sensitivity to light and sound, which are also commonly reported following SRC [11]. By advising these individuals to not participate, we aimed to ensure that reported symptoms were more likely attributable to SRCs, thereby enhancing internal validity and supporting more reliable interpretation of associations. The initial stakeholder outreach was multinational in scope, encompassing countries in North America (Canada, United States), Europe (United Kingdom, Turkey), and Oceania (Australia, New Zealand).

The study aimed to recruit as many participants as possible; however, several challenges constrained this approach. First, it was not possible to control how widely stakeholders disseminated the questionnaire or the extent to which athletes chose to participate and further share the survey. Consequently, a true sampling frame could not be established *a-priori*. Second, the analysis prioritised estimates of lifetime prevalence and odds ratios with 95% compatibility intervals rather than p-values, recognising the potential value of these data for future meta-analyses or meta-regressions. Indeed, even studies with small samples can contribute meaningfully to pooled analyses with greater statistical power, particularly in sports such as volleyball, which have received limited research attention. Third, the study was conducted as part of a dissertation in Sport and Exercise Medicine, which necessitated a constrained data collection window to allow timely completion of the degree. Additionally, resources for translation beyond English and Turkish were not available, limiting the potential to widen the sampling frame.

### Data Collection

A non-validated questionnaire was developed in English and subsequently translated into Turkish by the researcher, a native Turkish speaker. The translation was then reviewed and validated by two bilingual healthcare professionals proficient in both Turkish and English to ensure accuracy and linguistic validity. Additionally, a pilot study was conducted with two volleyball players who met the inclusion criteria. They reviewed the survey questions and provided feedback on clarity and comprehensibility. Based on their feedback, minor terminology revisions were made. No data were collected from the pilot study participants, and they did not participate in the main survey.

Both questionnaires were developed using the latest version (v3) of the Joint Information Systems Committee (JISC) survey tool and were available between May and July 2024. The aim was to provide descriptive insight into current views and practices rather than generate quantitative scores or assess psychometric properties. The questionnaire consisted of five sections. The first two sections included the participant information sheet and consent form. Section 3 gathered demographic information, such as age, sex (at birth), playing position, playing level, and playing experience. Subgroups were defined to reflect meaningful differences in volleyball participation and exposure. Sex (at birth) was categorised as male or female, consistent with standard sport and volleyball-specific research [5,7,8,19]. Playing level included high school/university, amateur, semi-professional, and professional, reflecting typical competition tiers. Playing position distinguished right/left hitter, centre hitter, setter, and libero, capturing the distinct roles and movement patterns associated with potential head impact exposure. Both factors were categorised to reflect differences in competition intensity, exposure to injury risk, and position-specific risks [5]. Age bands (16–21, 22–27, 28–33, 34–39, 39 + years) were selected to capture transitions from late adolescence into early adult competitive phases, mid-career, and later career, during which recovery capacity, life commitments, injury reporting, risk-taking behaviours, and injury risk may vary [8,9]. Playing experience categories (1–3, 4–8, 9–13, 13 + years) were chosen to reflect early integration into the sport, skill levels, higher training and competition exposure, accumulation of playing experience, and long-term participation, all of which may influence exposure, injury risk, and reporting behaviour. Although reasoned, these categories are pragmatic and exploratory, intended for descriptive stratification rather than validated thresholds. Section 4 provided information on the current definition of SRC, as outlined at the 6th International Conference on Concussion in Sport [11], along with potential symptoms following a head impact. It then asked about SRC history, including injury mechanisms, activity type, and the phase of the season during which the injury occurred. Section 5 examined players’ immediate medical attention, follow-up care, post-concussion syndrome, and return-to-sport decisions. A copy of the questionnaire can be found in S1 File.

### Statistical analysis

Data were transferred from the JISC server to Microsoft Excel (Version 2409), and data cleaning procedures were implemented to identify and address missing or inconsistent entries. The data were then imported into the Statistical Package for the Social Sciences (SPSS, Version 31, Armonk, USA) to estimate lifetime prevalence and assess associations between key contextual factors and reporting SRC.

Descriptive statistics and cross-tabulations were used to summarise sample characteristics. Lifetime prevalence of SRC was calculated using the formula: $\frac{Number\;of\;participant\;with\;SRC}{Total\;number\;of\;participant\;in\;each\;category}\;x\;\text{1}\text{0}\text{0}$. A narrative description was also provided on the incidences of SRC, medical support, injury mechanisms, activity type, season phases, SRC consequences (e.g., time loss, missed matches, persisting post-concussion symptoms), and return-to-sport decision-making.

To assess the association between sex at birth, age group, playing experience, playing level, and playing position (fixed factors) with the odds of reporting SRC (dependent variable), a mixed-effect binary logistic model was performed at both univariable and multivariable levels. A repeated-measures structure was used to account for those reporting multiple instances of SRC. A series of univariable models were constructed to assess the individual association between each fixed factor and the dependent variable. This was followed by multivariable regression analysis, with SRC as the dependent variable (yes vs. no) and personal characteristics (age, gender, playing experience, playing level, and playing position) as fixed factors. The results of this analysis presented the odds ratio (ORs) and corresponding 95% compatibility intervals. Interpretation of the odds ratio was based on Hopkins [20] and the reciprocals: trivial (0.99 to 0.68 and 1.01 to 1.49), small (0.67 to 0.30 and 1.50 to 3.49), moderate (0.29 to 0.12 and 3.50 to 8.99), large (0.11 to 0.29 and 9.00 to 31.90), and very large (0.03 and 32.0). P-values are presented as absolute values to supplement interpretation.

## Results

A total of 91 volleyball players completed the questionnaire. 17 were excluded from the final analysis due to unreliable results, as evidenced by inconsistencies in the responses. Of the 74 players included in the final analysis, 25 experienced a total of 51 SRCs with the remaining players indicated they had not experienced an SRC in the past (*n* = 49). The lifetime prevalence was 35%. The athletes were categorised into the following age groups: 16–21 years (n = 26), 22–27 years (n = 19), 28–33 years (n = 11), 34–39 years (n = 9), and 39 years and older (n = 9). A full breakdown of participants who experienced an SRC or not is presented in Table 1, including the associated subgroup prevalence.

**Table 1:** Personal characteristics and the prevalence of SRC.

		Self-reported SRC		Prevalence
		Yes (n)	No (n)	
Sex	Male	10	20	33%
	Female	16	28	36%
Age (years)	16-21	7	19	27%
	22-27	3	16	16%
	28-33	6	5	55%
	34-39	4	5	44%
	39+	7	3	70%
Playing experience (years)	1-3	4	13	24%
	4-8	3	13	19%
	9-13	4	10	29%
	13+	15	12	56%
Playing league	High school/ University	7	23	23%
	Amateur	16	15	52%
	Semi-professional	2	6	25%
	Professional	1	4	20%
Playing position	Right/ Left Hitter	8	19	30%
	Centre Hitter	6	9	40%
	Setter	6	13	32%
	Libero	6	7	46%

When considering the total number of incidents, the season phase indicated that the majority of concussions occurred during competition (45.1%), with the remaining 35.3%, 11.8%, and 7.8% occurring during in-season training, pre-game preparations, and pre-season training, respectively. Digging was the activity type most frequently associated with SRC (37.3%), followed by general play (29.4%), blocking (19.6%), and passing (15.7%). Conditioning activity (2.0%), serving (2.0%), spiking (2.0%), and other non-game activities (5.9%) accounted for the remainder. The leading cause of injury was ball-to-head contact, accounting for 70.6% of cases. Additionally, player-to-player contact and ground contact accounted for 23.5% and 13.7% of injuries, respectively.

**Fig 1:**
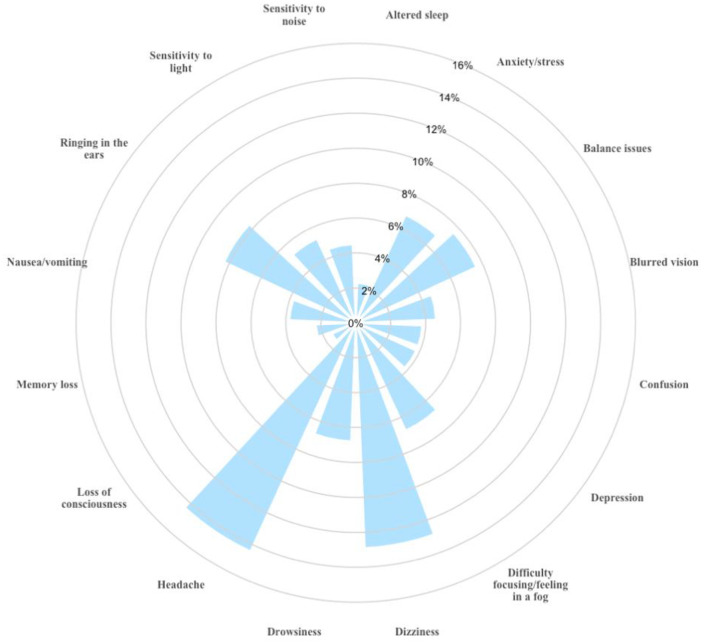
Frequency of self-reported symptoms experienced following a SRC.

Among those who experienced an SRC, headache was the most commonly reported symptom (14.4%), followed by dizziness (12.9%), ringing in the ears (8.3%), and balance problems (7.6%). Less frequent symptoms, ranging from 3.8% to 6.8%, included confusion, depression, memory loss, nausea, anxiety, fatigue, difficulty focusing, sensitivity to light, double vision, and noise sensitivity. Loss of consciousness was rare (1.5%) (Fig 1).

Most players who experienced an SRC did not seek or receive medical support during or after the incident, nor did they undergo a full medical assessment. As such, 61% were not given a formal diagnosis and only 10% underwent rehabilitation after the injury. A large proportion of players did not have any rest time or time away from matches. Despite nearly half reporting that they did not feel recorded before returning to play, 60% received no medical support beforehand (Table 2).

**Table 2:** Summary of medical care, recovery, implications on training and return-to-sport data among athletes (*n* = 26) who self-reported 51 SRCs.

		Self-reported SRC	
		No. SRCs	Percentage
Received medical help when the incident occurred	No	39	77%
	Yes	12	23%
Received medical help after the incident occurred	No	37	73%
	Yes	14	27%
Received doctor’s examination after the injury occurred	No	35	69%
	Yes	16	31%
Received physical rehabilitation programme afterwards	No	46	90%
	Yes	5	10%
Diagnosed SRC	No	31	61%
	Yes	20	39%
Resting time	None	25	49%
	<1 week	8	16%
	1-2 weeks	12	24%
	>3 weeks	6	11%
Missed matches	0	34	67%
	1-3	13	25%
	4-8	4	8%
Medical support during return to sport decision making	Sports Medicine Physician	6	12%
	General Practitioner	4	8%
	Neurologist	2	4%
	Athletic Trainer/ Coach	8	16%
	No one	31	60%
Feeling fully recovered before return-to-sport	No	26	51%
	Yes	25	49%

*Note: No SRCs = the total number of SRCs reported by 26 athletes.*

**Fig 2:**
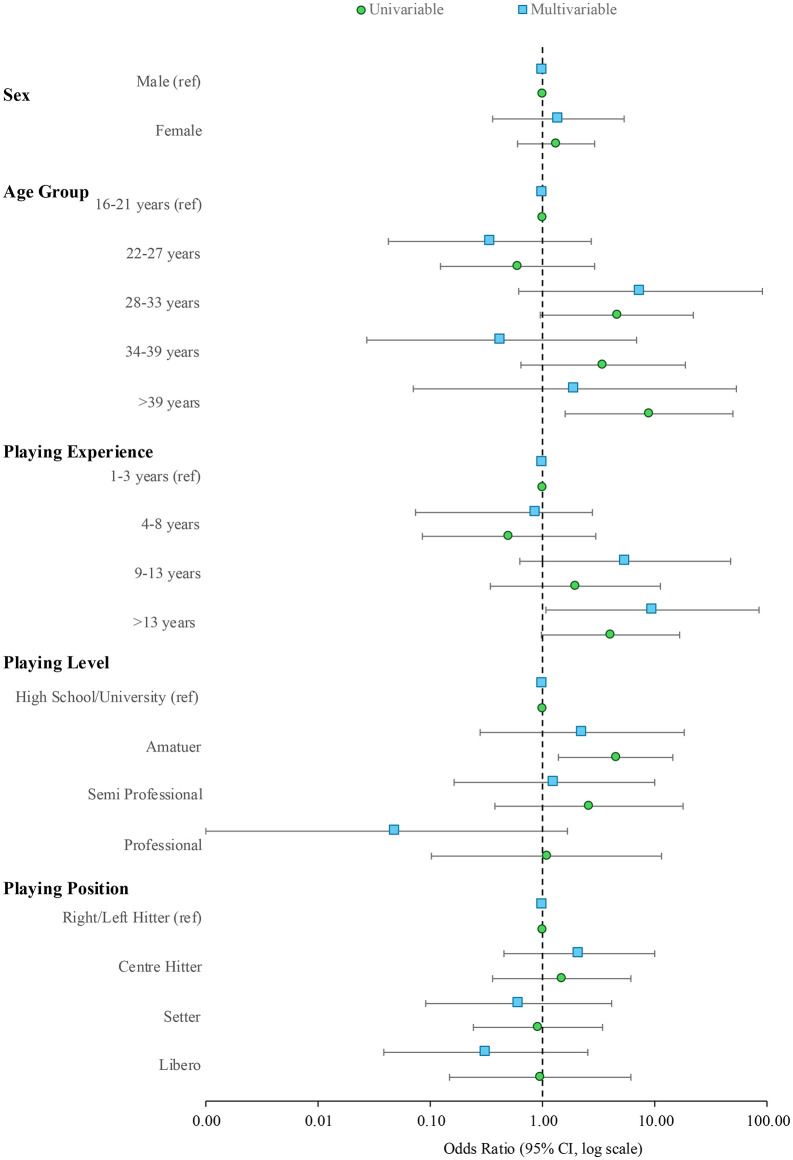
Association between sport-related concussions and sport-related factors including personal characteristics.

The mixed-effect binary logistic regression included 101 data points from 74 participants, with point estimates ranging from trivial to moderate and many including wide compatibility intervals that crossed negative, null, and positive associations (Figure 2, Table 3). Positive associations were observed for the 28–33-year age group, and for those with over 13 years of playing experience at both the univariable and multivariable levels, compared to those aged 16–21 years and with one to three years of experience, respectively. Those playing at an amateur level, and those aged 34 to over 39 years, also showed greater odds of reporting an SRC compared with high school or university players, although this was only evident at a univariable level. At a multivariable level, those aged 22–27 years, and those categorised as professional, showed reduced odds of reporting a SRC. The point estimates for playing position showed greater odds in centre hitters and reduced odds in setters and liberos, although the compatibility intervals ranged from positive to negative.

**Table 3:** Univariable and multivariable binary logistic regression analysis of sport-related factors and personal characteristics.

	Univariable Model				Multivariable Model			
		95% CL				95% CL		
Variable	OR	Lower	Upper	*P*	OR	Lower	Upper	*P*
Gender								
Male (ref)	–	–	–	–	–	–	–	–
Female	1.32	0.60	2.93	0.49	1.39	0.36	5.33	0.64
Age								
16–21 years (ref)	–	–	–	–	–	–	–	–
22-27 years	0.60	0.12	2.91	0.52	0.34	0.04	2.72	0.31
28-33 years	4.58	0.96	21.94	0.06	7.34	1.61	90.72	0.12
34-39 years	3.44	0.64	18.53	0.15	0.43	0.03	6.84	0.55
39+ years	8.91	1.58	50.18	0.01	1.92	0.07	52.71	0.70
Playing Experience								
1–3 years (ref)	–	–	–	–	–	–	–	–
4-8 years	0.50	0.09	2.95	0.44	0.86	0.07	2.81	0.39
9-13 years	1.97	0.34	11.32	0.45	5.45	0.62	47.91	0.13
13+ years	4.04	0.97	16.74	0.05	9.51	1.06	85.31	0.04
Current League								
High School/University (ref)	–	–	–	–	–	–	–	–
Amateur	4.48	1.38	14.51	0.01	2.25	0.28	18.16	0.45
Semi Professional	2.60	0.38	17.86	0.33	1.27	0.16	9.87	0.82
Professional	1.08	0.10	10.50	0.95	0.05	0.00	1.66	0.09
Playing Position								
Right/Left Hitter (ref)	–	–	–	–	–	–	–	–
Centre Hitter	1.48	0.36	6.16	0.59	2.11	0.45	9.90	0.34
Setter	0.91	0.24	3.44	0.89	0.61	0.09	4.08	0.61
Libero	1.95	0.15	6.07	0.96	0.31	0.04	2.53	0.28

Ref = referent or reference group. OR = odds ratio 95%CL = 95% compatibility intervals. P = probability.

## Discussion

This study explored the self-reported lifetime prevalence of SRC and the nature of post-injury behaviour among volleyball players. The findings indicate a lifetime prevalence of 35%, with limited post-injury help-seeking behaviour around diagnosis, advice, initial care, prescribed rest, time away from play, and return-to-sport management following SRC. Increased odds of reporting a SRC were associated with being female, aged 28–33 years, having more than nine years of playing experience, competing at amateur or semi-professional levels, and playing in the centre or libero positions. Conversely, reduced odds of SRC were observed among players aged 22–27 years, those with four to eight years of playing experience, and those in the setter position.

Our first aim was to estimate the self-reported prevalence of SRC in volleyball among athletes recruited from multiple countries. Our results indicated a prevalence of 35%, with some variation between males and females, suggesting that approximately one in three volleyball players may experience a SRC over their lifetime of participating in the sport. Considering global participation numbers, this would translate to a substantial number of SRC cases worldwide. In England and the United States, where participation estimates are available, this prevalence could equate to approximately 30,000 and 2.3 million cases, respectively. This rate exceeds previous reports for youth and college-level volleyball [10,12,21]. However, differences in sampled populations and observation or recall periods must be considered. For instance, Veliz et al. [21] reported prevalence among 8th, 10th, and 12th grade students over the preceding 12 months. Our findings align with Daugherty et al. [22], who reported a 29.8% prevalence in a large sample of American adults using varying definitions of concussion, and McCallum et al. [23], who found a similar lifetime prevalence of 28.6% among high school students.

Our data indicate that competitive match-play contributes to a higher prevalence of SRC, with 56.9% occurring during a competitive event (pre- and in-game) compared to 43.1% in training, potentially due to the strategic nature and intensity of matches, which may involve higher risk-taking. This finding contrasts with previous reports on collegiate-level players [2]. The discrepancy between our study and previous research may be due to the latter focusing on collegiate players within specific seasons, whereas our sample includes all league levels and lifetime self-reported SRC data. Moreover, it is likely that players spend significantly more time training than competing; however, this study did not capture training and match volume due to the cross-sectional design and use of recall. These findings underscore the need for longitudinal studies to further examine SRC incidence rates in training and competition, as well as the impact of competitive play on SRC risk across all playing levels. Such studies will ensure a comprehensive understanding of SRC risk factors that can inform future risk-reduction efforts. In our study, ball-to-head contact accounted for 70.6% of SRCs, aligning with previous findings ranging from 57.1% to 61.5% [2,9,10,12]. This high prevalence can be attributed to the high velocity of serves and spikes, which reduce players’ reaction times and increase the likelihood of a direct impact to the head.

Digging emerged as a risk factor in this study, accounting for over one-third of SRCs, and has been reported as a leading cause of injury among women in NCAA competitions [2]. Zuckerman et al. [13] found that surface contact during digging was the most common activity-mechanism combination for SRC. This finding agrees with the work of Reeser et al. [24], who found just over half of all SRCs in high school and college occurred whilst executing the digging action; player-to-surface contact explained 13.7% to 37.3% of all SRCs. It may also be that the digging action, which requires players to adopt a crouched position, increases their susceptibility to accidental contact with teammates’ knees or hips, further elevating the risk. General play ranked second, highlighting the need for further investigation into why routine movements and transitions-those not associated with specific skills such as digging, spiking, or blocking-might carry increased SRC risk. A possible explanation is that structured actions like digging, spiking, and blocking rely on learned motor patterns and anticipatory responses, whereas general play involves more reactive movements requiring quicker reaction times, which may increase the risk of an impact to the head or a whiplash-like action. Understanding these factors can help develop targeted preventive measures, including structuring general play, improving reaction time, incorporating neck-strengthening exercises, and enhancing head-awareness training, especially for liberos who frequently engage in digging.

Our second aim was to understand the post-SRC responses of players across the 51 reported cases. In relatively few instances, medical help was sought or received during or after the incident, which may explain why approximately two-thirds of SRCs went undiagnosed. Additionally, responses related to rest, the number of missed games, decision-making around return to play, and symptom resolution suggest that volleyball players may resume activity while still experiencing signs and symptoms, without receiving medical clearance from a healthcare professional. This is concerning given the potential risk that comes from subsequent concussions [25]. Other possible explanations for the lack of diagnosis include limited access to medical professionals [26], particularly at amateur levels where healthcare provision is not as easily available as in professional contexts; poor recognition of signs and symptoms by coaches and athletes [27–28]; club culture and external pressures that may discourage reporting [29–30]; and knowledge and attitudes towards SRC, which can influence willingness to report [31–33]. Regulatory bodies may consider providing guidance and policy to ensure recommendations are in place and widely available at all levels of volleyball to facilitate athlete safety.

Our third aim was to investigate the association between personal and sport characteristics and SRC, focusing on sex, age, playing experience, playing level, and position. Sex differences in SRC risk have recently gained attention in volleyball research. Previous studies suggest that disparities may stem from physical and physiological differences, such as neck strength, which is associated with head accelerations during impact [34–37]. In this study, we found no meaningful difference in the odds of reporting SRC between male and female volleyball players. It is important to note, however, that these results reflect self-reported SRC. Nonetheless, these findings suggest there is little evidence of sex differences in volleyball, which may reflect that the sport is typically considered a limited-contact sport, thought to influence between-sex prevalence [38] and agrees with some, [39,40] but not all studies [38] of varying populations.

Regarding age, participants aged 28–39 years showed higher odds of reporting a SRC in the univariable model. However, in the multivariable model, this pattern remained only for the 28–33 years and 39 years and older age groups. For those aged 34–39 years, the direction of the effect changed between the univariable and multivariable models, suggesting some instability in the estimate or that the effect is explained by other factors included in the model. A similar observation was seen for those with 9–13 years’ experience and those with more than 13 years’ of experience, who demonstrated higher odds of reporting a SRC in both univariable and multivariable analyses. Older and more experienced athletes likely represent players at their competitive peak, with sustained exposure to higher training and match demands, frequent involvement in high-risk actions such as blocking, digging, and spiking, and a greater accumulated opportunity to sustain and recognise SRCs over time [2,10,13]. This pattern was less apparent for those groups closer to the reference group (i.e., 22–27 years and 4–8 years), though slightly lower odds were observed from the point estimates among players aged 22–27 years and with 4–8 years of playing experience. While caution is needed here due to the wide compatibility intervals that encompass positive and negative associations as well as encompass a null assoication, this may reflect lower cumulative exposure to elite match demands, fewer years experiencing the sport at higher competitive levels, or reduced likelihood of recalling or reporting previous SRC incident. Notably, elite-level volleyball competition (e.g., Olympic Games) often features athletes with a mean age above 27 years for both males and females [41], suggesting that older and more experienced athletes may represent a high-risk subgroup. It could also be that those within these groups are most likely to report incidents of SRC within this study, while those at an elite level are more likely to have incidents noted within medical records than other groups. Nonetheless, further research is required to elucidate how age and experience influence concussion risk and reporting in volleyball.

In our multivariable model, professional players demonstrated substantially lower odds of reporting SRC compared with high school and university athletes. Although the compatibility interval crossed the null, indicating uncertainty in the direction of effect, it suggests a potentially protective association at the professional level. This may reflect the advanced technical proficiency, physical conditioning, and refined movement control characteristic of elite performers, including superior trunk and core strength [42], which collectively support safer landing strategies and controlled defensive movements that minimise exposure to inadvertent head impacts. In addition, professional environments often provide comprehensive medical oversight, structured concussion education, and established return-to-participation protocols, which may enhance SRC injury prevention practices. Differences in reporting behaviour and contextual incentives may also contribute. This pattern contrasts with previous literature in which collegiate athletes have demonstrated greater SRC risk relative to high school athletes, with one study reporting a 3.4-fold increase in collegiate settings [24]. Finally, positional differences were less pronounced than in previous studies (e.g., [2]), with all data reflecting positive, null, and negative associations. Prior research has identified middle hitters as particularly susceptible to SRC, possibly due to their frequent involvement in both offensive (spiking) and defensive (blocking) actions. Larger-scale, longitudinal research is needed to explore and confirm positional risk patterns in volleyball, as this may have implications for equipment use and policy, training and technical development, and the medical care of these athletes with regard to potential future SRCs.

This study also has some limitations that are worth noting. Firstly, the relatively small and unequal sample distribution regarding sex, playing league, and position from a self-selected population results in low statistical power and wide compatibility intervals, as well as reduced external validity of these findings, requiring caution when interpreting and applying these results. We are also unable to comment on the response rate due to not knowing how many participants had access to the study’s advertising material, and acknowledge this may have resulted in self-selection bias. Secondly, a reliance on self-reported concussion history introduces potential recall bias. Thirdly, we are unable to confirm accurately which countries the responses originated from, meaning the true external validity of this work is unknown when considering potential country-specific variation. Finally, we note that a non-validated questionnaire was used, given the exploratory nature of the study, rather than attempting to quantify knowledge, attitudes, or reporting behaviours. Future research is required to confirm the findings of this study using a larger sample, limiting the recall period, collecting data longitudinally, and exploring the reasons behind participants’ responses. Therefore, the results of this study should be considered exploratory and require confirmation through future large-scale cross-sectional and cohort studies.

### Conclusion

This study provides exploratory, novel insight into the self-reported lifetime prevalence of SRC among volleyball players, highlighting a relatively high rate of 35%, with digging and ball-to-head contact identified as common mechanisms. The lack of medical intervention and structured management following SRC raises concerns about athlete safety and the need for improved recognition, reporting, and access to care. Our findings suggest that SRC risk may be partially influenced by multiple factors, including age, playing experience, level, and position, though associations were generally small and should be interpreted with caution due to sample limitations and wide compatibility intervals. Overall, these findings support the need for sport-specific, context-sensitive strategies to reduce SRC risk in volleyball and point to the importance of future longitudinal studies to confirm these results and inform prevention and education efforts across all levels of play.

## Supporting information

S1 File Questionnaire Revised. (DOCX)

S2 File Dataeset Revised. (XLSX)

S3 File Human Subject Checklist Revised. (DOCX)
