# Supplementary material for: Self-reported concussion prevalence, post-injury help-seeking behaviour, and associated risk factors among volleyball players
Source: PLoS One. 2025 Dec 5;20(12):e0338225. doi: 10.1371/journal.pone.0338225 (PMC12680157; doi:10.1371/journal.pone.0338225)
Supplement: S1 File — (DOCX) [file pone.0338225.s001.docx]

**S1- QUESTIONNAIRE**

**PARTICIPANT INFORMATION SHEET**

head impact and concussion in volleyball: effect of playing level, playing position, timeline of injuries, and sex

**1. Invitation to take part**

I would like to invite you to participate on the ‘Head Impacts and Concussion in Volleyball’ research project. My name is Gamze Nedzhipoglu, and I am a master's degree student in Manchester Metropolitan University.

My research project aims to determine the prevalence and factors associated with sports-related concussion in the volleyball community by using a cross-sectional survey. This research project is not funded by any organisation.

**2. Why have I been invited?**

I seek participants who can contribute to the research by joining the survey. I am contacting you because you are a volleyball players who competed at any level of the game for at least one year and are aged 16 years and older. Taking part in the study is voluntary.

**3. Do I have to take part?**

It is up to you to decide. We will describe the aims of the study and all aspects involved in this participant information sheet. The next page will include a consent form. You will be asked to read each statement and tick each box if you agree to the statement. At the end of the survey you will be asked to confirm that you consent before clicking “submit” . You are free to withdraw at any time without giving a reason by simply closing your browser. Once you have clicked the final submit button you will be unable to withdraw as this questionnaire is fully anonymous.

**4. What will I be asked to do?**

Once you have read this information sheet and agree to the consent statements, you will be asked to provide your age and gender which we will included as factors that might influence reporting sports-related concussion. Additionally, you will be asked about your involvement in volleyball and with concussion. Specifically, we will ask you about your playing level and position, duration on the team, history of concussions, frequency of such injuries, and whether these incidents led to a temporary withdrawal from sports. The survey aims to investigate the prevalence and factors affecting concussion in volleyball through the evaluation of participant responses. The project ensures participant anonymity. No personal data that can identify you is needed for this study.

**5. Are there any risks if I participate?**

No risks are anticipated in this study given it’s a fully anonymous online survey. That said, we do highlight that experiences of sports-related concussion can be a traumatic event to recall. Therefore, if at any point you feel any form of distress, we would recommend you pause the survey and leave it fully if needed. If you do become distressed or worried, we recommend contacting your local health care provided.

There is no penalty or sanction for withdrawing from the study.

The results of the research project will be shared in the journal if it is published. Since the results will be fully anonymous, you will not be identifiable.

**6. Are there any advantages if I participate?**

Even though there are no direct compensations or rewards for participating in this study, your input is important to better understand concussion in volleyball.

**7. What will happen with the data I provide?**

The Manchester Metropolitan University (‘the University’) is the Data Controller in respect of this research and any personal data that you provide as a research participant. The University is registered with the Information Commissioner’s Office (ICO) and manages personal data in accordance with the General Data Protection Regulation (GDPR) and the University’s Data Protection Policy.

When you agree to participate in this research, we will collect personal data as part of this research (such as gender, age, profession). As a public authority acting in the public interest, we rely upon the ‘public task’ lawful basis. When we collect special category data (such as medical information) we rely upon the research and archiving purposes in the public interest lawful basis.

Your rights to access, change or move your information are limited, as we need to manage your information in specific ways in order for the research to be reliable and accurate. Withdrawal from the study can be conducted during the survey. Withdrawal from the study after submitting the survey will not be taken into account.

We will only retain your personal data for as long as is necessary to achieve the research purpose, which we estimate to be one year after the completion of the research project, due in October 2025. Your personal information will be kept separately, in a securely encrypted database, from the contents of your actual participation. All of the digital material will be kept encrypted on the institutional server. The consent sheet will be kept locked within the MMU facilities. We will not share your survey responses collected in this form with any third parties.

After the period mentioned above, your personal data will be destroyed as follows: digital data will follow the Her Majesty’s Government Infosec Standard 5 Enhanced UK; physical paper data will be Micro Crosscut shredded.

For further information about use of your personal data and your data protection rights please see the [University’s Data Protection Pages](https://www2.mmu.ac.uk/data-protection/).

**What will happen to the results of the research study?**

The results of the study will be made publicly available through submission to a scientific journal. Results will also be presented in academic conferences on Health and Medicine. Results will be published in English.

**Who has reviewed this research project?**

This research project has been reviewed and approved by the Faculty Research Ethics & Governance Committee (FREGC) of Manchester Metropolitan University.

**Who do I contact if I have concerns about this study or I wish to complain?**

If you would like more information about the study, please contact Ms Gamze Nedzhipoglu ([GAMZE.NEDZHIPOGLU@stu.mmu.ac.uk](mailto:GAMZE.NEDZHIPOGLU@stu.mmu.ac.uk)).

If you would like to raise any concerns, please contact Dr Amanda Johnson ([M.johnson@mmu.ac.uk](mailto:M.johnson@mmu.ac.uk)).

Any complaints should be directed to the Faculty of Health and Education Research Ethics and Governance Committee ([FOHE-ethics@mmu.ac.uk](mailto:FOHE-ethics@mmu.ac.uk)).

If you have any concerns regarding the personal data collected from you, our Data Protection Officer can be contacted using the [legal@mmu.ac.uk](mailto:legal@mmu.ac.uk) e-mail address, by calling 0161 247 3331 or in writing to: Data Protection Officer, Legal Services, All Saints Building, Manchester Metropolitan University, Manchester, M15 6BH. You also have a right to lodge a complaint in respect of the processing of your personal data with the Information Commissioner’s Office as the supervisory authority. Please see: <https://ico.org.uk/global/contact-us/>

**THANK YOU FOR CONSIDERING PARTICIPATING IN THIS PROJECT**

**CONSENT FORM**

|  | Please tick your chosen answer | I agree |
| --- | --- | --- |
| 1 | I confirm that I have read the participant information sheet version 1.9, date 18/04/2024 for the above study. | ☐ |
| 2 | I have had the opportunity to consider the information, ask questions and have had these answered satisfactorily. | ☐ |
| 3 | I understand that my participation is voluntary and that I am free to withdraw at any time without giving any reason, without my legal rights being affected. | ☐ |
| 4 | I agree to participate in the project to the extent of the activities described to me in the above participant information sheet. | ☐ |
| 5 | I give permission for a fully anonymised version of the data I provide to be deposited in an Open Access repository so that it can be used for future research and learning. | ☐ |

**QUESTIONNAIRE**

1. What is your sex?
2. What is your age?
3. How many years have you played volleyball?
4. How many years have you played volleyball?
5. Which league are you currently playing in?
6. Which position are you currently playing on the field?
7. Have you been diagnosed with migraine or post-traumatic stress disorder?
8. Have you ever experienced the following symptoms after a head impact during your involvement in volleyball? Please choose according to your symptoms: Confusion, Anxiety/stress, Ringing in the ears, Focusing problems/mental fogginess, Feeling tired/drowsy, Sensitivity to light, Depression, Loss of consciousness (even brief), Dizziness, Headache, Nausea/vomiting, Memory loss, Balance problems, Sensitivity to noise, Changes in sleep patterns, Double/blurry vision, Other, None of the above?
9. How many concussions have you had throughout your career that occurred during your involvement in sports?
10. How many of your concussions occurred within a six-month period?
11. How many times have you been diagnosed with a concussion after being examined by a healthcare professional?
12. In which league were you playing when you had a concussion? If you have had more than one concussion and were in different leagues during them, please choose all that apply.
13. What was your field position when you had a concussion? If you had more than one concussion and held different positions during them, please choose all that apply.
14. In which phase of your training or competition schedule did the concussion occur? If you have had more than one concussion occurring in different occasions, please choose all that apply.
15. What activity were you engaged in when the concussion occurred? If you have had more than one concussion that occurred during different sport activities, please choose all that apply.
16. What was the mechanism of injury for your concussion? If you have had multiple concussions with different injury mechanisms, please select all that apply.
17. How many of your concussions received medical help when they occurred?
18. How many of your concussions received medical help after occurring?
19. How many of your concussions received a doctor’s examination after the injury occurred?
20. How many of your concussions received a physical rehabilitation programme afterwards?
21. How many matches did you miss after a concussion occurred? If you have had more than one concussion with a different number of missed matches, please choose all that apply.
22. How many week did you rest after your concussion? If you have had more than one concussion and the injuries resolved in different weeks, please choose all that apply.
23. How many of your concussion injuries have been examined by healthcare professionals before returning to sports?
24. Whose decision was it to approve your return-to-sport? If you have had more than one concussion and the return-to-sport decision was made by different professionals, please choose all that apply.
25. How many of your concussion injuries did you feel fully recovered from before returning to sport?
26. Post-concussion syndrome is lasting concussion symptoms for more than a 3-month period. How many times have you experienced post-concussion syndrome after a concussion injury?
