# Supplementary material for: Self-reported concussion prevalence, post-injury help-seeking behaviour, and associated risk factors among volleyball players
Source: PLoS One. 2025 Dec 5;20(12):e0338225. doi: 10.1371/journal.pone.0338225 (PMC12680157; doi:10.1371/journal.pone.0338225)
Supplement: S3 File — (DOCX) [file pone.0338225.s003.docx]

**Human Participants Research Checklist**

***Complete the following if your study involved human participants or human participants’ data. These questions should be addressed for prospective and retrospective studies.***

1. Did you obtain ethics approval for this study?
   - If yes, please upload (file type “Other”) the original approval document you received from your ethics committee. If the original document is in another language, please also provide an English translation.

An approval letter has been uploaded. Please note that this work stems from a student project whereby approval was granted by both the project supervisor (Dr Amanda Johnson) and an independent reviewer on behalf of the Faculty of Health and Education Research Ethics and Governance Committee at Manchester Metropolitan University.

- - If you did not obtain ethical approval, please explain why this was not required below.

1. If you prospectively recruited human participants for the study – for example, you conducted a clinical trial, distributed questionnaires, or obtained tissues, data or samples for the purposes of this study, please report in the Methods:
   1. the day, month and year of the **start and end** of the recruitment period for this study.
   2. whether participants provided informed consent, and if so, what type was obtained (for instance, written or verbal, and if verbal, how it was documented and witnessed). If your study included minors, state whether you obtained consent from parents or guardians. If the need for consent was waived by the ethics committee, please include this information.

Recruitment and data collection began on the 1st of June 2024 and end on the 28^th^ of August 2024. This information is provided on line 159-160.

Consent was obtained via an electronic consent form embedded into the questionnaire. In the UK, those aged 16 are not considered minor hence being set as the inclusion criteria. Approval for this was granted by our institution ethics committee. This information is on line 177.

1. If you are reporting a retrospective study of medical records or archived samples, please report in the Methods section:
2. the day, month and year when the data were accessed for research purposes
3. whether authors had access to information that could identify individual participants during or after data collection

Not applicable
